# High-density genetic map construction and comparative genome analysis in asparagus bean

**DOI:** 10.1038/s41598-018-23173-0

**Published:** 2018-03-19

**Authors:** Haitao Huang, Huaqiang Tan, Dongmei Xu, Yi Tang, Yisong Niu, Yunsong Lai, Manman Tie, Huanxiu Li

**Affiliations:** 1 College of Horticulture, Sichuan Agricultural University, Chengdu, 611130 China; 2 Mianyang Academy of Agricultural Sciences, Mianyang, 621023 China; 3 Dazhou Academy of Agricultural Sciences, Dazhou, 635000 China

## Abstract

Genetic maps are a prerequisite for quantitative trait locus (QTL) analysis, marker-assisted selection (MAS), fine gene mapping, and assembly of genome sequences. So far, several asparagus bean linkage maps have been established using various kinds of molecular markers. However, these maps were all constructed by gel- or array-based markers. No maps based on sequencing method have been reported. In this study, an NGS-based strategy, SLAF-seq, was applied to create a high-density genetic map for asparagus bean. Through SLAF library construction and Illumina sequencing of two parents and 100 F2 individuals, a total of 55,437 polymorphic SLAF markers were developed and mined for SNP markers. The map consisted of 5,225 SNP markers in 11 LGs, spanning a total distance of 1,850.81 cM, with an average distance between markers of 0.35 cM. Comparative genome analysis with four other legume species, soybean, common bean, mung bean and adzuki bean showed that asparagus bean is genetically more related to adzuki bean. The results will provide a foundation for future genomic research, such as QTL fine mapping, comparative mapping in pulses, and offer support for assembling asparagus bean genome sequence.

## Introduction

Asparagus bean [*Vigna unguiculata* (L.) Walp. ssp. *sesquipedalis*] (2n = 2x= 22) is one of the two main cultivated subspecies of cowpea. Its common names include yardlong bean, Chinese long bean, snake bean, pea bean, and so on^1^. Unlike its counterpart, the *unguiculata* group (ssp. *unguiculata*), which is widely grown in Africa, India, Middle East and the Americas for dry grain^2^, asparagus bean is widely cultivated in Southeast and East Asia for its immature pods as a vegetable^3^. Hence, asparagus bean is also called ‘vegetable cowpea’. Apart from geographic location and consuming organ, asparagus bean also differs from the *unguiculata* group in pod length, plant type, and growth habit^4^. This special legume serves as an important source of dietary proteins, vitamins, minerals, and fibers for Asian people^5^ and is considered one of the top ten Asian vegetables.

Genetic maps are related to modern plant biology and offer a foundation for downstream studies, such as quantitative trait locus (QTL) mapping, marker-assisted selection, and fine gene mapping. Additionally, those maps can be helpful for the assembly of genome sequence. The last two decades have witnessed the advancement of genetic maps in cowpea. The first map was constructed by Fatokun *et al*.^6^, which is based on RFLP markers in an F2 population of 58 individuals derived from a cross between IT 2246-4 and TVNI 963. After that, several cowpea linkage maps have been established using AFLP^7^, RAPD^7,8^ and SSR markers^2,9,10^.

For these traditional gel-based markers, due to their high cost, long cycle, and low success rate, it is difficult to obtain the required number of markers for a high-resolution map. Of all the marker types, SNP (single nucleotide polymorphism) has become the first choice in many evolutionary and ecological studies. Because SNP is the most abundant and stable genetic variation in the genome^11^. Not long ago, three maps based on SNP markers generated from GoldenGate platform were constructed^2,12,13^. More recently, two high-density maps were also created by SNP markers from the Cowpea iSelect Consortium Array^14,15^. Along with the advancement of molecular marker techniques applied in cowpea maps, the density of these maps has been dramatically increased to 0.1 cM^14^. However, all the five SNP cowpea maps so far are all based on SNP array. No maps that reply on sequencing-based technology have been reported.

NGS (Next Generation Sequencing) technologies are imposing a substantial impact on many areas of biology, including the analysis of genetic diversity in populations, genetic map construction and QTL mapping. Although the cost of sequencing is plummeting, sequencing the entire genome of every individual in a population is still very expensive. In fact, when using polymorphisms to answer some biological questions, only a small part of genomic regions need to be measured^16^. RRL (Reduced Representation Library) is a method which samples and sequences a subset of genome regions instead of sequencing the whole genome. The use of RRLs for SNP discovery was first described using Sanger sequencing^17^: pools of DNA from multiple individuals were completely digested by restriction endonuclease, then through the size selection of fragments, the complexity of DNA had been reduced. Specific length amplified fragment sequencing (SLAF-seq) is an enhanced RRL sequencing method^18^. SLAF technology is improved in three aspects: i) Pre-design scheme for SLAF selection using training data. ii) SLAF-seq library construction. iii) Deep sequencing for the pooled RRLs, and genotype definition and validation by software. The efficiency of this method had been demonstrated by considerable genotyping accuracy on rice and soybean data, as well as the highest density genetic map for common carp^18^. More importantly, it has already been proved to be a powerful tool to create high-density genetic maps in quite a few species^19–29^, even those without reference genome.

In a previous study, we had conducted a transcriptome analysis between two asparagus bean cultivars^30^. One of the cultivars, Dubai bean, is cold tolerant, while the other one, Ningjiang 3, is cold sensitive. We constructed an F2 population stemmed from a cross between the two varieties. The objective of this study was to: i) create a high-density genetic map for asparagus bean using an NGS-based strategy, SLAF-seq; ii) perform comparative genome analysis with other widely cultivated and consumed legumes using obtained SLAF markers.

## Results

### SLAF sequencing

Trough massively high-throughput sequencing of SLAF libraries, a total of 68.43 Gb of raw data were generated, which consisted of 342,168,780 pair-end reads with 100 bp in length (Table 1). The percentage of high quality reads with a score of Q30 (a quality score of 30, indicating 0.1% sequencing error rate) was more than 94% and the average guanine-cytosine (GC) content was 40.98%.

**Table 1:** Basic statistics of the SLAF-seq data in asparagus bean.

Sample ID	Total Reads	GC percentage (%)	Q30 percentage (%)
Dubai bean (Paternal line)	11,711,940	40.30	94.55
Ningjiang 3 (Maternal line)	11,166,372	40.31	94.59
Average of 100 offspring	3,192,905	40.17	94.37
Rice (Control)	912,829	41.16	94.13
Total	342,168,780	40.98	94.37

After sequence alignment and clustering, the numbers of SLAFs detected in the male parent and female parent were 356,174 and 343,311, with an average depth (the average number of reads in each SLAF) of 27.19-fold and 27.41-fold, respectively. In the Offspring, i.e., the F2 population, the number of SLAFs ranged from 165,895 to 300,831 with an average value of 223,623 and a mean depth of 12.38-fold. Overall, 785,712 SLAFs were obtained, among which 55,437 were polymorphic, indicating a polymorphism rate of 7.05%.

### SNP Markers

187,555 SNPs were mined from the 55,437 polymorphic SLAF markers, of which 187,119 biallelic SNPs were used for further analysis. These SNP markers were preliminarily screened and grouped. Those lacking parent information, less than 4-fold in depth or not polymorphic in two parental lines were excluded. The number of remaining SNPs was 18,634 (Table 2), which were classified into four segregation patterns (aa × bb, hk × hk, lm × ll, nn × np). Since the two parental lines are fully homozygous with aa or bb genotype, only the aa × bb segregation pattern was chosen for linkage map construction, which consisted of 13,257 SNP markers. The other three segregation types, lm × ll, nn × np, hk × hk, represented markers heterozygous only in male parent ‘Dubai bean’, only in female parent ‘Ningjiang 3’, and in both parents, respectively. Afterwards, these markers were further filtered based on three criteria: i) Markers with depths <10-fold in the parents and <1-fold in offspring were removed. ii) The integrity of the markers (the percentage of genotyped markers of the total markers in each individual) should be >75%. iii) Markers with significant segregation distortion (P < 0.01) resulted from a chi-square test were excluded. Eventually, 5,380 SNP markers were selected for genetic map construction.

**Table 2:** The whole process of filtering SNP markers.

Type	Number
Total_marker	187,119
Parent marker lack	136,902
Depth not meet	24,327
Nopoly marker	7,256
Remain_marker	18,634
aaxbb	13,257
hkxhk	2,295
lmxll	1,584
nnxnp	1,498
aaxbb_final	5,380

### Genetic mapping

**Figure 1:**
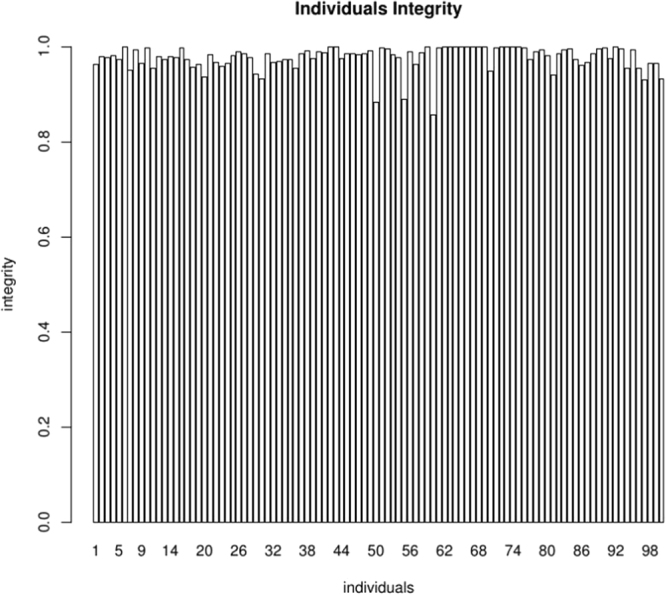
The marker integrity in each individual. The x-axis stands for progeny code and the y-axis corresponds to marker integrity.

Altogether, 5,380 SNPs were loaded into HighMap software. Through linkage analysis, 5,225 SNPs (97.21%) were anchored to the map, distributing over 11 linkage groups (LGs). Information of these SNP markers and their corresponding SLAF marker sequences can be found in Supplementary Tables S1 and S2. The average depth of these markers were 46.39-fold for the male parent, 44.81-fold for the female parent, and 13.28-fold for each F2 individual. The average integrity of the mapped markers was 97.62%, which indicated a relatively high map quality (Fig. 1).

**Figure 2:**
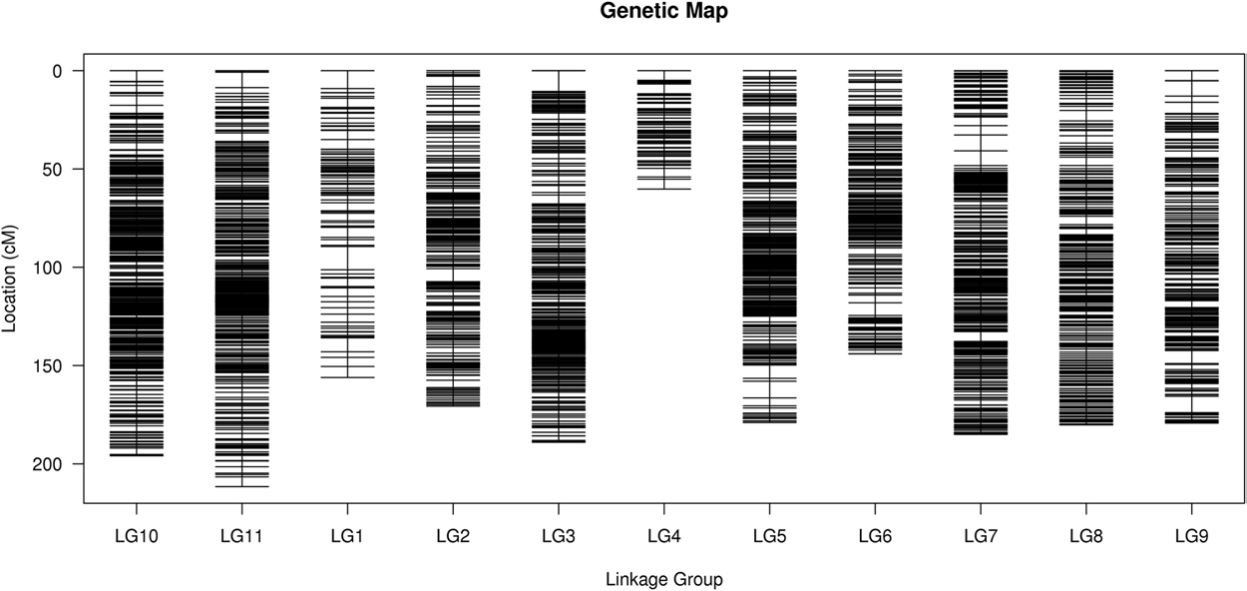
Distribution of SNP markers on 11 linkage groups. A black bar indicates a SNP marker. Linkage group number is shown on the x-axis, and genetic distance (cM) is shown on the y-axis.

The final genetic map spanned a total of 1,850.81 centi Morgan (cM), with an average distance of 0.35 cM between adjacent markers (Table 3). In addition, Fig. 2 shows that the largest LG is LG11. It has 684 SNP markers on a total distance of 211.57 cM with an average value of 0.31 cM. The smallest LG, meanwhile, is LG 4 that has only 89 markers. The total distance is merely 60.23 cM and the mean value is 1.37 cM. The percentage of Gap <5 cM ranges from 95.61% in LG1 to 99.85% in LG11, with a mean figure of 99.09%. Another important parameter that reflects the degree of linkage between markers, Max Gap in each LG, fluctuates between 5.04 cM in LG4 and 11.82 cM in LG1, with an average of 7.63 cM.

**Table 3:** Basic information of asparagus bean genetic map.

Linkage Group ID	Total Marker	Total Distance (cM)	Average Distance (cM)	Gap <5 cM	Max Gap (cM)
LG1	115	156.07	1.37	95.61%	11.82
LG2	367	170.58	0.47	99.45%	6.6
LG3	545	189.02	0.35	99.26%	10.55
LG4	89	60.23	0.68	98.86%	5.04
LG5	515	178.97	0.35	99.61%	8.51
LG6	383	144.02	0.38	99.74%	6.48
LG7	653	185.04	0.28	99.54%	8.06
LG8	870	180.19	0.21	99.77%	5.32
LG9	390	179.27	0.46	98.46%	8.31
LG10	614	195.85	0.32	99.84%	5.45
LG11	684	211.57	0.31	99.85%	7.85
Average	475	168.26	0.35	99.09%	7.64
Total	5225	1850.81	—	—	—

### Map rating

The quality of the asparagus bean map was assessed by creating the haplotype and heat maps. A haplotype map mirrors the double crossover of the population caused by genotyping errors. The majority of recombination blocks were clearly defined (Supplementary Fig. S1). The recombination rate was relatively low and The proportion of missing markers for each LG was less than 2.55% (Table 4).

**Table 4:** The recombination rate and missing percent of all the linkage groups.

Linkage Group	Recombination Rate (%)	Missing Percent (%)
1	3.15	2.39
2	0.41	1.92
3	0.27	1.05
4	2.40	2.55
5	0.24	0.6
6	0.31	1.26
7	0.28	0.35
8	0.01	0.14
9	0.22	1.02
10	0.36	1.44
11	0.35	0.82

Heat maps are able to reflect the recombination relationship between markers in each LG and identify the potential ordering errors. Consequently, heat maps were also generated by using pair-wise recombination values for the 5,225 mapped SNPs, which showed that these markers in most LGs were well ordered (Supplementary Fig. S2).

### Comparative genome analysis

**Figure 3:**
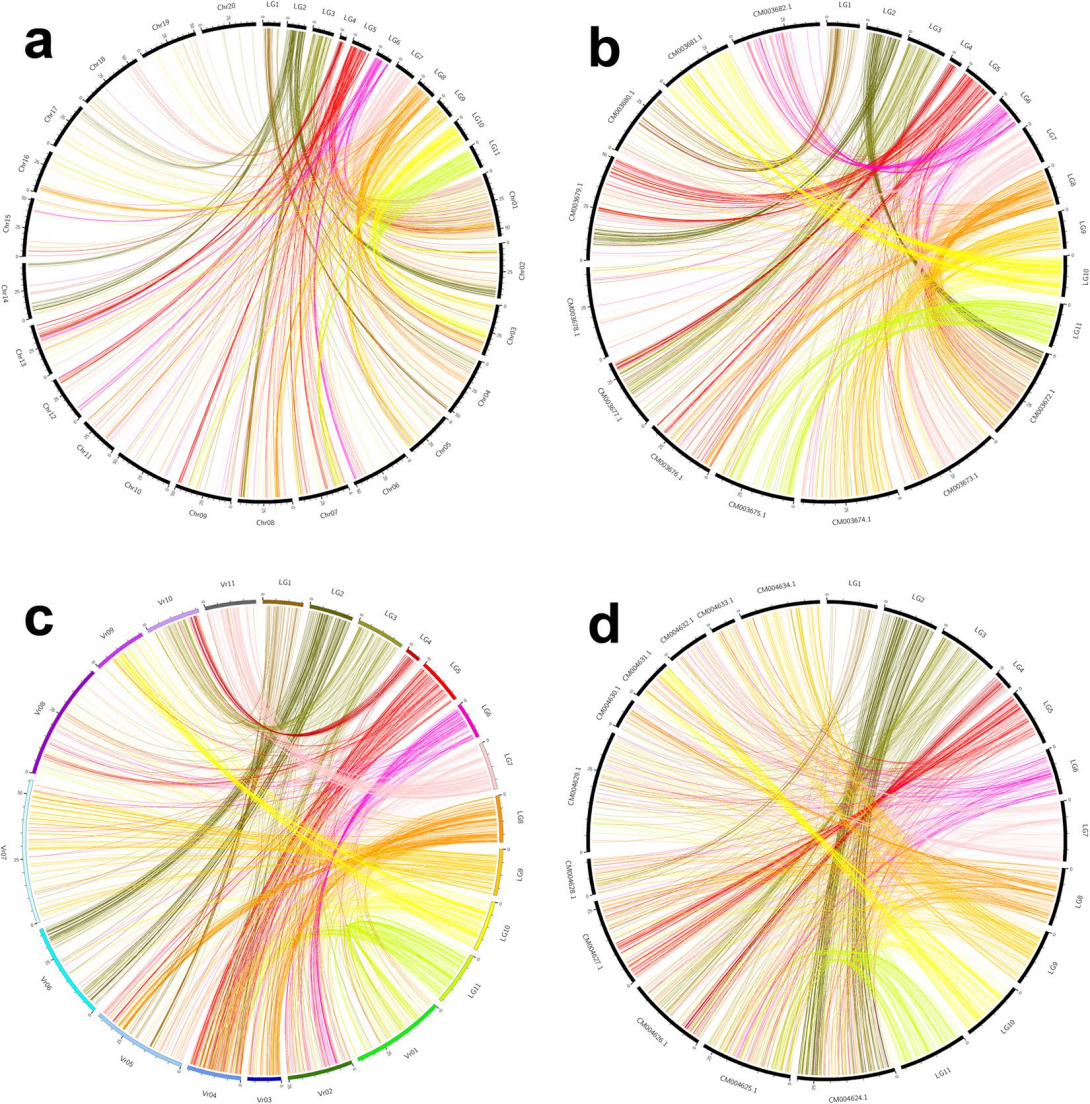
Comparison between asparagus bean and four other pulse genomes. Circos plots show linear relationship between asparagus bean and soybean (**a**), common bean (**b**), mung bean (**c**) and adzuki bean (**d**). LG1 to LG11 represent the 11 LGs in asparagus bean. Others symbolize chromosomes of each legume.

The 785,712 SLAF markers generated from asparagus bean were used for comparative analysis with the genome sequences of four other legume species: soybean (*Glycine max*), common bean (*Phaseolus vulgaris*), mung bean (*Vigna radiata*), and adzuki bean (*Vigna angularis*). Of the 785,712 SLAF markers, the numbers matching the genome of these four species were 14,291 (1.82%), 56,046 (7.13%), 52,175 (6.64%) and 80,472 (10.24%), respectively (Table 5). The 3,554 mapped SLAF makers, which contained SNP markers used for linkage map construction, were also assessed in these four pulse species. The data showed a similar trend to that of total markers, with 1,022 (28.76%) matching to soybean, 1,581 (44.49%) matching to common bean, 1,881 (52.93%) matching to mung bean, and 2,044 (57.51%) matching to adzuki bean. These results indicated that asparagus bean is more closely related to adzuki bean than other three legumes. A Circos plot was produced to demonstrate a correspondence between the mapped SLAF markers and their genomic locations in soybean, common bean, mung bean, and adzuki bean (Fig. 3), visualizing linear relationships between asparagus bean and these four pulses. The four plots illustrated that the mapped SLAF markers located in each of the 11 LG were also distributed across different chromosomes of the four legumes. Since the genome sequence of asparagus bean is not available at the moment, the relationships between asparagus bean and other legume species that have reference genome are extremely useful for asparagus bean researchers.

**Table 5:** Comparative genome analyses between asparagus bean and four other pulse species.

	Total SLAF tags	Percentage	Mapped SLAF makers	Percentage
Total	785712		3554	
Soybean	14291	1.82%	1022	28.76%
Common bean	56046	7.13%	1581	44.49%
Mung bean	52175	6.64%	1881	52.93%
Adzuki bean	80472	10.24%	2044	57.51%

## Discussion

Selection of parental material is the first step in preparing a mapping population. Dubai bean is a variety introduced from Dubai, the United Arab Emirates. It is cold tolerant and characterized by small and brown seeds, dark green and short pods. While Ningjiang 3 is a widely grown cultivar in Sichuan province and other parts of China mainly because of its high yield (Supplementary Fig. S3). However, it is sensitive to low temperature and has bigger and dark seeds, light green and much longer pods. Therefore, the two cultivars are largely different in seed color and size, pod hue and length, the number of pods produced, as well as cold tolerance, which will facilitate QTL analysis of these important traits in the future, because basically pods and seeds of asparagus bean are the consuming organ for humans.

For genetic research purpose, hand or artificial pollination is necessary. However, cowpea is cleistogamous, producing viable pollens and receptive stigma before blossom. This phenomenon imposes entirely self-pollination on the crop^31^. The success of artificial pollination has been reported to be low, ranging from 0.5 to 50%. Thus, in order to make sure the two parental lines were successfully hybridized, several morphological traits related to seeds and pods of their offspring were examined. The seed color of F1 was dark, and the size seemed to be bigger than both parents (Supplementary Fig. S4). Also, the progeny had long and dark green pods (Supplementary Fig. S5), which meant they inherited the length from the female parent and color from the male parent. These characters proved that the hybridization was successful, laying a solid foundation for further analysis.

With the massively parallel NGS technology, high-throughput identification and genotyping of SNP markers become realistic. Although the cost of sequencing is plummeting, whole-genome deep sequencing is still cost-prohibitive for large populations. One of the strategies that reduce the costs of sequencing is called reduced representation library (RRL) sequencing, which reduces the complexity of the genome. SLAF-seq and RAD-seq^32^ (Restriction-site Associated DNA sequencing) are two representative RRL sequencing methods that simplify the genome. RAD-seq is a method that samples at reduced complexity of target genomes, producing high resolution population genomic data for any organism at low costs. In comparison with RAD, SLAF technique makes use of pair-end reads, double barcodes and most importantly, a pilot experiment before sequencing, which ensures the density, uniformity and efficiency of the marker development. It has also been reported that SLAF repeatability is better than RAD^22,25^. Moreover, reference genome sequences are not necessary for this method. To summarize, SLAF-seq is a fast, accurate, highly efficient, and cost-effective strategy for de novo SNP discovery and genotyping of large populations^18,24^. SLAF-seq is able to develop more than 5,000 SNP markers for map construction and the average distance between markers can be lower than 0.5 cM^20,23,25,29^.

According to some published research, the genetic diversity of cowpea is relatively low^33–35^. Accessions of self-pollinated crops such as cowpea often possess low within accession variability^36^. The narrow genetic base results in a low ratio of polymorphic bands generated from traditional markers^37–40^, which makes it impossible to construct a high-resolution genetic map in cowpea. Traditional markers can only increase the density of cowpea map to 3 cM^10^. Compared with conventional markers, SNP markers are becoming more and more popular in plant breeding duo to their high-throughput. By using SNPs, the map density has been greatly improved. Murcho *et al*.^13^ developed an Illumina1536-SNP GoldenGate genotyping array from nearly 10,000 high confidence EST-derived SNPs. Based on this platform, a cowpea consensus genetic map was constructed from six mapping populations, which consists of 928 SNP markers, spanning 680 cM with 11 LGs and an average marker distance of 0.73 cM. Afterwards, an improved consensus map was constructed from 13 population-specific maps^12^. This consensus map contained 1107 EST-derived SNP markers on 11 linkage groups. The average distance was 0.79 cM between bins. On average, there is one bin per 733 Kb of the cowpea genome. Unfortunately, by applying this array to an asparagus bean map, only 191 SNPs were detected and mapped instead of about 1000 SNPs^2^. This equals to a low percentage of nearly 20%. Since the mapping parents have distant geographic origin and different phenotype, this supports the point that the genetic variation in asparagus bean is lower than in ssp. *unguiculata*, which is consistent with several previous genetic diversity studies^2,34,41^.

Recently, a more advanced platform, the Cowpea iSelect Consortium Array was developed^15^, which includes 51,128 SNPs derived from WGS sequencing of 37 diverse cowpea accessions. This is a wonderful tool to genotype a mapping population and a large association panel for cowpea to map QTLs and facilitate data exchanging between research programs. Based on this platform, Munoz-Amatrian *et al*.^15^ constructed a consensus map from five cowpea RIL populations, which contains 37,372 SNP loci mapped to 3,280 bins. The map spans 837.11 cM at an average density of one bin per 0.26 cM. Using the same method, Xu *et al*.^14^ constructed a high-density map for asparagus bean. 7,988 SNPs were mapped to 697 bins in 11 LGs. The map length is 803.4 cM with an average distance of 1.15 cM between bins or 0.1 cM between markers.

The five maps above are all constructed based on array. However, array-based genotyping is relatively inflexible, as the SNPs on an array cannot be added or replaced easily. Therefore, array-based genotyping is prone to ascertainment bias when analyzing samples from different gene pools^42^. Also, markers on the genetic map may be unevenly distributed^14^. In addition, although the Cowpea iSelect Consortium Array is developed from 37 diverse cowpea accessions, it only includes four asparagus bean accessions from China^15^. In this study, since Dubai bean is introduced from another country, i.e. the United Arab Emirates. Its genetic background is unknown. Thus, a sequencing-based method, SLAF-seq, was chosen, which ensures the density, uniformity and efficiency of marker development. 187,555 SNPs were mined from polymorphic SLAF markers and, after filtering, 5,225 SNPs were used for genetic map construction. The map consists of 11 LGs, which correspond to the haploid number of chromosomes of asparagus bean. It spans a total distance of 1,850.81 cM, almost two to three times as long as the maps previously reported by Muchero *et al*.^13^ (680 cM), Xu *et al*.^2^ (745 cM), Lucas *et al*.^12^ (680 cM), Munoz-Amatrian *et al*.^15^ (837.11 cM) and Xu *et al*.^14^ (803.4 cM), rendering it the second longest map for cowpea available to date (the most extensive cowpea map is 2670 cM, created by Ouedraogo *et al*.^7^). The average distance between markers is 0.35 cM, or one SNP per 118 kbp considering the cowpea genome to be 620 Mbp. The density is not as high as the previous asparagus bean map^14^ and the cowpea consensus map^15^, but much higher than another three SNP maps based on GoldenGate platform^2,12,13^. Besides, markers with significant segregation distortion (P < 0.01) resulted from a chi-square test were excluded for this map construction. If these markers were added later as accessory markers, the density could be higher. To gauge the quality of the map, haplotype map and heat map were created. The results showed that most of the LGs were uniformly distributed, and these markers in most LGs were well placed. Additionally, marker integrity and accuracy were high, suggesting that the genetic map was of high quality. Therefore, SLAF-seq is an alternative approach to construct high-density map for asparagus bean.

In the present study, SLAF-seq technology was applied in asparagus bean for the first time. A high-density genetic map with high quality was obtained, which is the first NGS-based map in asparagus bean. This high-resolution map will provide a wonderful platform for QTL fine mapping and molecular breeding in asparagus bean. In addition, this study has developed massive SNP markers for comparative genomic studies and future marker-assisted selection (MAS) in asparagus bean breeding program. They can also be applied on cultivar and hybridized offspring identification as well as genetic diversity analysis among cultivars. Moreover, since whole genome references of asparagus bean are not available, the sequence and location information of these markers, which were explored at the whole genome level, could be served as a reference data for genome assemblies of asparagus bean in the future.

## Conclusion

In this study, a high-density map of asparagus bean was constructed. To the best of our knowledge, this is the first NGS-based linkage map for this pulse. Comparative genome analysis was also performed, which showed that asparagus bean is more related to adzuki bean than soybean, common bean and mung bean. The results will provide an excellent platform for future genomic research, such as mapping QTLs for other important traits, fine gene mapping, comparative mapping in legumes, and will offer support in the process of assembling the asparagus bean genome sequence.

## Methods

### Plant materials and DNA extraction

In 2015, two cultivars, Dubai bean and Ningjiang 3, were hybridized to generate F1 population at Mianyang Academy of Agricultural Science Research, Mianyang, Sichuan, China (30°42′N, 105°43′E, 500 m above sea level). The area features a subtropical monsoon climate and is largely mild and humid, with four distinct seasons. The male parent, Dubai bean, is introduced from Dubai, the United Arab Emirates, and is a cold tolerant material. While the female parent, Ningjiang 3, is a landrace widely grown in Sichuan Province and some other parts of China but is sensitive to low temperature^26^. Then F1 seeds were sown in the same year and selfed to generate F2 population because this pulse can be cultivated and harvested twice a year in this region. F2 seeds and the two parents were planted in April of 2016. A subset of 100 F2 individuals and the two parents were used as mapping population. Young leaves were collected and genomic DNA was isolated following the CTAB protocol^43^. The concentration and quality of DNA were determined by a NanoDrop ND-1000 spectrophotometer (Thermo Fisher Scientific, MA, USA). DNA integrity was confirmed by electrophoresis on 1% agarose gel.

### SLAF library construction and high-throughput sequencing

A subset of 100 F2 individuals and the two parents were subjected to SLAF library construction and sequencing. The procedures were carried out according to Sun *et al*.^18^ with minor modifications. Since the whole genomic sequence of cowpea is not available so far, the genome of *Vigna radiata* was chosen as the reference for *in silico* simulation of restriction enzyme digestion. Based on the result of simulation, HaeIII and Hpy166II were finally chosen for genomic DNA digestion. Then an A-tail was added to each of the digested fragments. Afterwards, these fragments were subjected to PCR to add barcode 1. The products were separated on a 2% agarose gel, and those ranging from 314 to 444 bp in size were excised and purified with a Gel Extraction Kit (Qiagen, Hilden, Germany) before adding barcode 2 through PCR amplification. Finally, the amplicons were isolated, purified and diluted for paired-end sequencing on a HiSeq^TM^2500 (Illumina, Inc., USA) according to the manufacturer’s protocol. In order to gauge the accuracy of SLAF library construction and prevent false positive reads, rice (*Oryza sativa* L. *japonica*) was used as control and to perform the same steps as mentioned above.

### Sequence data grouping and genotyping

Low-quality reads (quality score <30) were deleted in the first place. Other reads were assigned to each offspring based on the duplex barcode sequence. Then these barcode sequences were trimmed, as well as the terminal 5-bp positions from each read. The reads obtained were called “clean reads”. All clean reads were clustered in accordance with sequence similarity detected by BLAT^44^. Sequences with over 95% identity were grouped in one SLAF locus. Cowpea is a diploid species, which means one locus may contain up to four SLAF tags, so groups containing more than four tags were considered as repetitive SLAFs and discarded. However, those with 2–4 tags were considered to be polymorphic SLAF markers. Subsequently, SNPs were detected from polymorphic SLAF markers using the software GATK^45^ and SAMtools^46^. These SNP markers were classified into four segregation patterns (hk × hk, lm × ll, nn × np and aa × bb). In the present study, the F2 population was obtained by selfing the F1 of a cross between two fully homozygous parents with a genotype of aa or bb. Therefore, only the SNPs with segregation patterns of aa × bb were employed for linkage map construction.

### Map construction

Finally obtained high-quality SNP markers were used for map construction using HighMap software^47^. HighMap consists of four modules, including linkage grouping, marker ordering, error genotyping correction and map evaluation. Firstly, in grouping module, markers were clustered into linkage groups by the single-linkage clustering algorithm, using a pair-wise modified independence LOD score as distance metric. Then an iterative process of marker ordering was performed by utilizing a combination of enhanced Gibbs sampling, spatial sampling and simulated annealing algorithms. After that, the SMOOTH algorithm^48^ was used to fix genotyping errors according to the parental contribution of the genotypes. The missing genotypes were imputed by a k-nearest neighbor algorithm^49^ and map distances in centi Morgan (cM) were estimated using the Kosambi mapping function^50^. At last, haplotype map and heat map were constructed, which would provide an intuitive and convenient way to rate map quality, with perl script “draw_haplotype-map.pl” and “draw_heatmap.pl”, respectively. They are programmed by Beijing Biomarker Technologies Corporation, and can be download at http://highmap.biomarker.com.cn/. The detailed approaches were according to Liu *et al*.^47^.

### Comparative genome analysis

The 785,712 SLAF markers generated from asparagus bean were aligned to the genome sequences of soybean (*Glycine max*), common bean (*Phaseolus vulgaris*), mung bean (*Vigna radiata*), and adzuki bean (*Vigna angularis*) using BLAST with e-value cutoff of ≤1e^−5^. Their GenBank assembly accessions were GCA_000004515.3, GCA_001517995.1, GCA_000741045.2, GCA_001723775.1, respectively.

### Data Availability

The SLAF-seq sequencing data generated during the current study has been deposited into NCBI Sequence Reads Archive (SRA) database with accession number SRP102460. The other data are included in this published article (and its Supplementary Information files).

## Electronic supplementary material

Supplementary Figures

Dataset 1
