# Supplementary material for: High-density genetic map construction and comparative genome analysis in asparagus bean
Source: Sci Rep. 2018 Mar 19;8:4836. doi: 10.1038/s41598-018-23173-0 (PMC5859152; doi:10.1038/s41598-018-23173-0)
Supplement: Supplementary file 1 — Supplementary Figures [file 41598_2018_23173_MOESM1_ESM.pdf]

High density genetic map construction and comparative genome analysis in asparagus bean

Haitao Huang[1,2,+], Huaqiang Tan[1,+], Dongmei Xu[2], Yi Tang[1], Yisong Niu[2], Yunsong Lai[1], Manman Tie[3], Huanxiu Li[1,*]

[1]College of Horticulture, Sichuan Agricultural University, Chengdu, 611130, China

[2]Mianyang Academy of Agricultural Science Research, Mianyang, 621023, China

[3]Dazhou Institute of Agricultural Sciences, Dazhou, 635000, China

[*]Corresponding author email: Huanxiuli62@gmail.com

[+]these authors contributed equally to this work

Huanxiu Li is the corresponding author and her information is as follows:

Department of Olericulture, College of Horticulture, Sichuan Agricultural University

Huimin Road No. 211, Wenjiang, Chengdu, 611130, Sichuan, China

Tel: +86-028-86291746

E-mail: Huanxiuli62@gmail.com

Running title: Asparagus bean genetic map and comparative genome analysis

Keywords: Asparagus bean, Genetic map, SLAF, Comparative genome

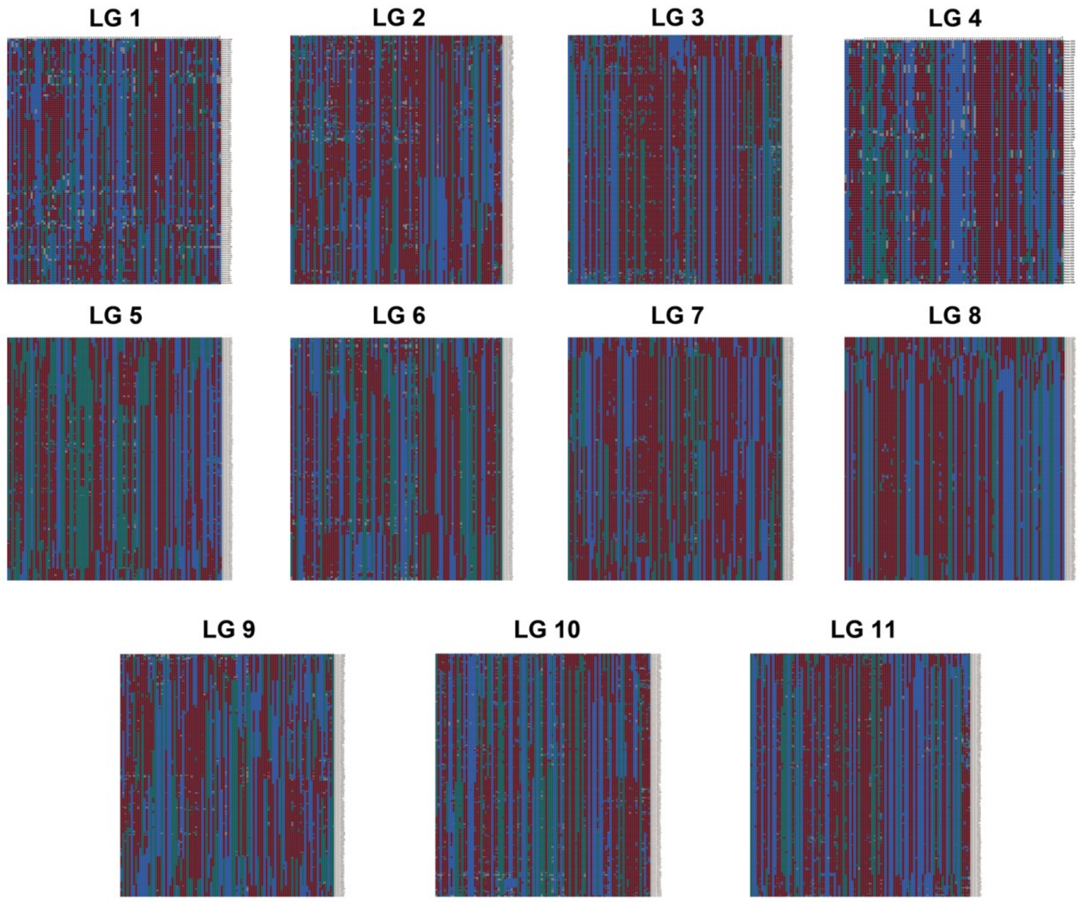

Supplementary Figure 1. Haplotype map of each LG. Each row represents a marker. Markers are arranged according to their position on each LG. Each column stands for one chromosome in an individual. A total of 100 columns mean 100 F2 individuals. Green corresponds to Ningjiang 3 (the female parent), blue symbolizes Dubai bean (the male parent) and red indicates heterozygosity. In the same column, the point at which the color changes is where recombinant event happens.

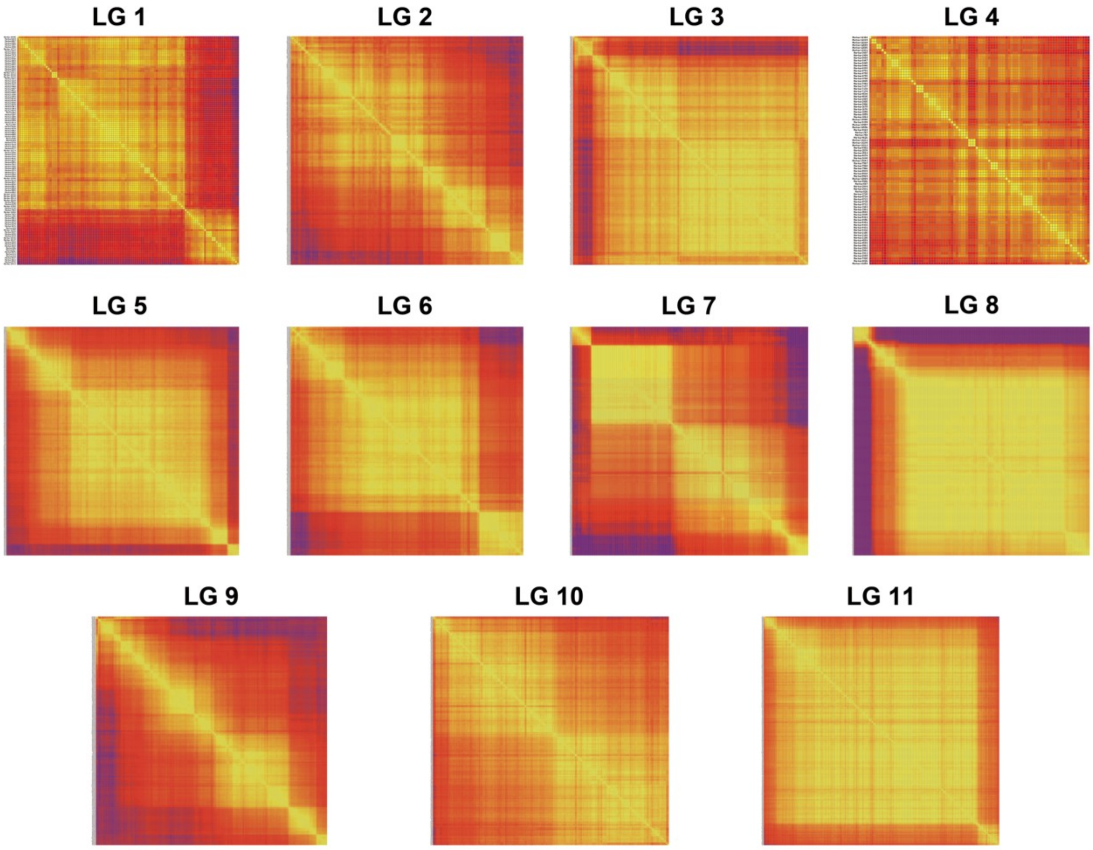

Supplementary Figure 2. Heat map of each LG. Each row and column are markers ranked according to their position on each LG. Each cell represents the recombination rate of two markers. Yellow indicates a lower recombination rate and purple a higher one.

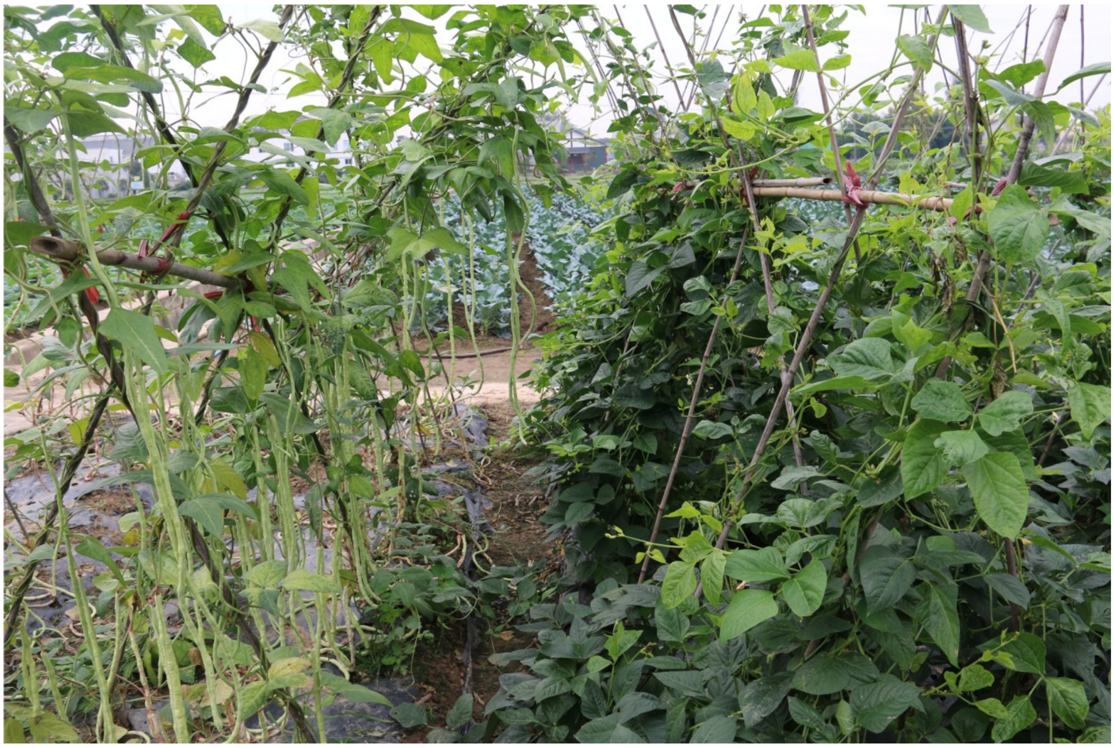

Supplementary Figure 3. The maternal line, Ningjiang 3 (left) and paternal line, Dubai bean (right). This picture was taken in October 16, 2016 at Mianyang Academy of Agricultural Science Research, Sichuan, China.

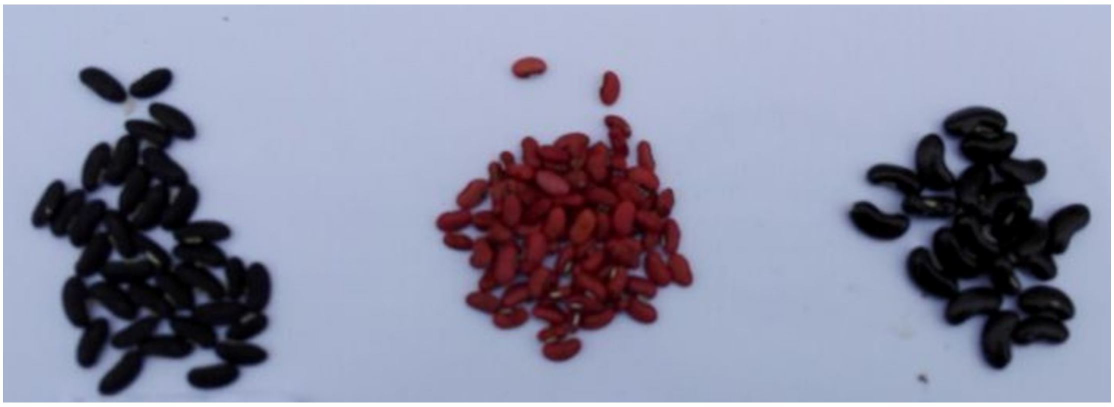

Supplementary Figure 4. The seeds of Ningjiang 3 (left), Dubai bean (middle) and F1 (right)

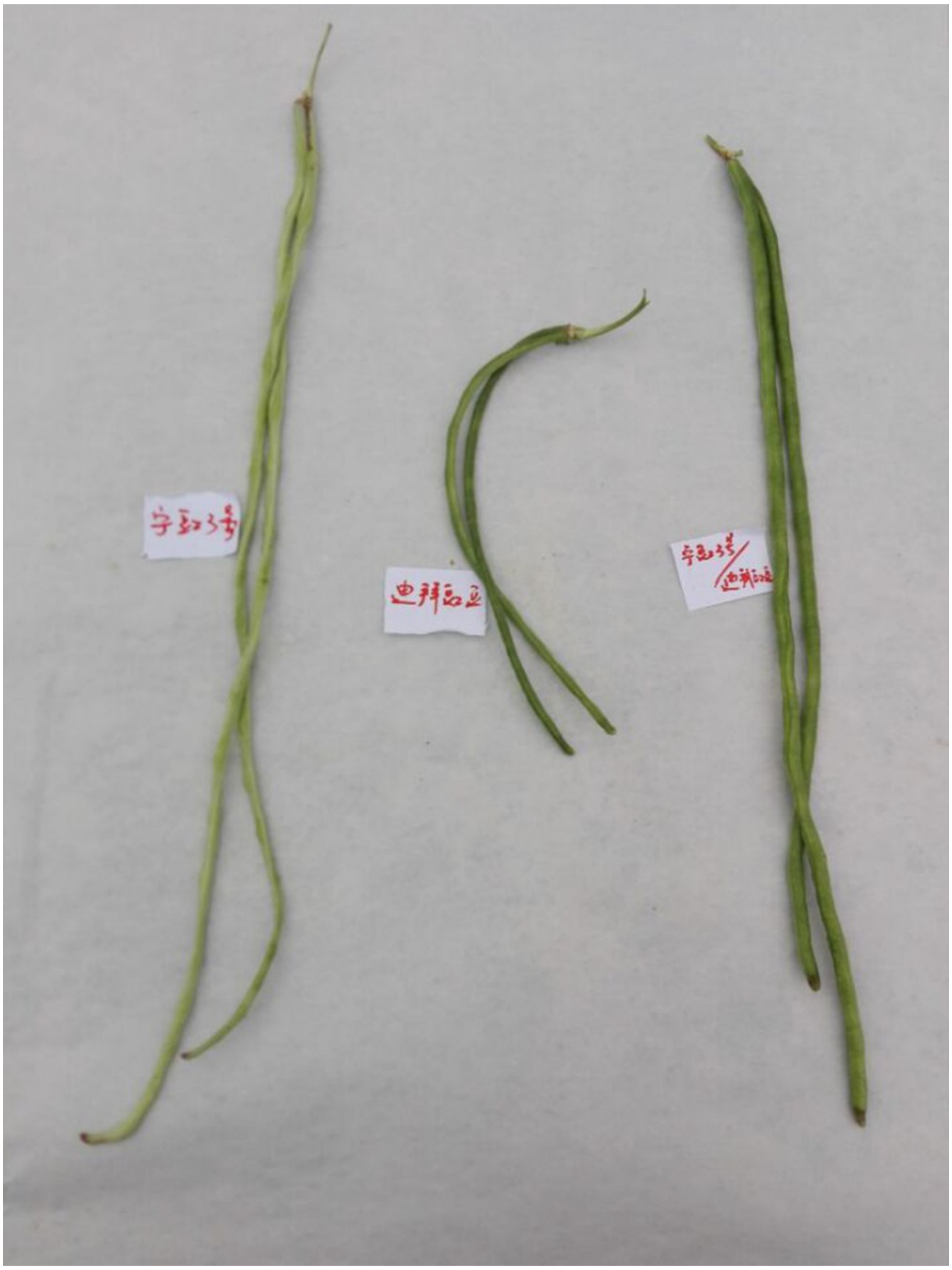

Supplementary Figure 5. The pod of Ningjiang 3 (left), Dubai bean (middle) and F1 (right)
